# Looking Back at My Years in Marine Natural Products Chemistry

**DOI:** 10.3390/md20030161

**Published:** 2022-02-24

**Authors:** Peter Proksch

**Affiliations:** Institute of Pharmaceutical Biology and Biotechnology, Heinrich-Heine-University Duesseldorf, 40225 Duesseldorf, Germany; proksch@uni-duesseldorf.de

When I started to work on marine natural products some thirty years ago I was attracted to this fascinating field of science by the exotic environment, the colourful shapes of (mostly) marine invertebrates and their complex ecological interactions. Having had mainly worked before on terrestrial plants the marine environment offered a totally new world for me, which has lost nothing of its thrill and fascination up to the present day. In parallel to marine macroorganisms I soon embarked on studying natural products from marine-derived microorganisms, which again opened up a new world both in terms of a huge biodiversity as well as highly unusual and complex microbial natural products, with many of them exhibiting pronounced biological activities. I consider myself very lucky for having been able to contribute to this field of science. The beauty of the marine environment and the thrill of treasure hunting for new and bioactive metabolites have never ceased to have a grip on me. 

Having said this, the key point of looking back at my years in marine natural products chemistry is, however, still missing. What continues to impress me the most are the friendships with collaborators, colleagues and students from all over the world whom I was lucky to meet along the way. Many of those have contributed to this Special Issue of *Marine Drugs*, which was dedicated to me and for which I am most grateful. Friendships that span thousands of kilometers and have already lasted for decades evolved from a mutual interest in marine natural products. These friendships, which included many collaborations with the home institutions of my former associates far away, have enabled me not only to embark on new projects but become acquainted with other cultures, other ways of living and other ways of looking at life. All of this has had a marked effect on my professional career and enriched my life tremendously. If someone were to ask me today what the most important aspect of my life as a scientist has been, my answer would be: meeting highly talented people along the way, helping them to embark on their own careers as scientists and finding friends for life. 

May this field of science never cease to attract young talents who help to push forward our knowledge of the sea and its hidden treasures.

